# Umbilically and Peripherally Inserted Thin Central Venous Catheters Have Similar Risks of Complications in Very Low-Birth-Weight Infants

**DOI:** 10.1177/00099228231161299

**Published:** 2023-03-21

**Authors:** S. Salonen, O. Tammela, A. M. Koivisto, P. Korhonen

**Affiliations:** 1 Faculty of Medicine and Health Technology, Tampere University, Tampere, Finland; 2 Tampere Center for Child Adolescent and Maternal Health Research, Faculty of Medicine and Health Technology, Tampere University Hospital, Tampere University, Tampere, Finland; 3 Faculties of Social Science and Health Science, Tampere University, Tampere, Finland

**Keywords:** catheter complication, catheter infection, peripherally inserted central catheter, thin umbilical catheter, very low birth weight infant

## Abstract

Catheter complications can be life-threatening in very low-birth-weight (VLBW) infants. We retrospectively evaluated non-elective removals of the first thin (1-2F) umbilical vein catheters (tUVCs (n = 92)) and peripherally inserted central venous catheters (PICCs (n = 103)) among 195 VLBW infants. Catheters were removed non-electively in 78 infants (40%), typically due to suspected infection (n = 42) or catheter dislocation (n = 30). Infants with complications had lower birth weights and gestational ages than others. The frequencies and causes of catheter removal were similar in the tUVC and PICC groups. Thirty-one infants had true catheter infections. The number of infections/1000 catheter days was higher in the tUVC group than in the PICC group. In a multivariable analysis, gestational age was associated with catheter infection, but catheter type was not. The odds of catheter complications decreased with increasing gestational age, but no clear association with thin catheter type was found.

## Introduction

Very low-birth-weight (VLBW) infants, who have birth weights of 1500 g or below, require time to achieve full enteral feeding, and parenteral nutrition may be necessary for many weeks. Central venous catheters (CVCs) ensure the administration of sufficient parenteral nutrition, fluids, and medication. However, the risk of complications associated with prolonged hospitalization, morbidity, and even death^1^ exists.

Thin peripherally inserted (1-2 F) central venous catheters (PICCs) have become increasingly common in the treatment of VLBW infants.^2^ The advantage of this catheter type is that they can be inserted and removed without anesthesia. Peripherally inserted central venous catheters are most commonly inserted into the veins of the upper extremities. By using the umbilical vein as a catheter route, painful skin penetration, which may also be associated with an increased risk of infection, can be avoided during the first days after birth. Traditional UVCs are recommended for use for only 4–10 days,^3^ whereas thin UVCs can be used for longer periods. The stiff properties of polyvinyl chloride may induce more vessel injury as compared with more-pliable silicone and polyurethane catheters. The use of polyurethane or polyvinyl chloride umbilical catheters has been associated with a significant incidence of thrombosis,^3-6^ and thus, their long-term use is inappropriate. Instead, thin silicone UVCs have been reported to have a low complication rate, one comparable with that of polyvinyl chloride umbilical catheters.^7^

The differences between PICCs and UVCs are controversial. Several studies have found no differences between PICCs and traditional UVCs in terms of general complication^2,8,9^ or catheter infection rates.^10,11^ Instead, one study found higher CVC-associated infection rates with umbilical catheters,^12^ and another detected a higher complication risk with PICCs as compared with umbilical catheters.^13^ Peripherally inserted central venous catheters and thin (2F) umbilically inserted silicone central venous catheters (tUVC) seem to pose an equal risk of thrombosis, catheter-related sepsis, and obstruction.^7^ More information is needed regarding the differences between thin UVCs and PICCs in VLBW infants.

Here, the aim was to investigate (1) the frequency and type of complications in the first thin CVCs; (2) the differences in catheter complications between tUVCs and PICCs; and (3) the predictors of catheter-related complications, especially catheter infections, in VLBW infants.

## Subjects and Methods

**Figure 1.**
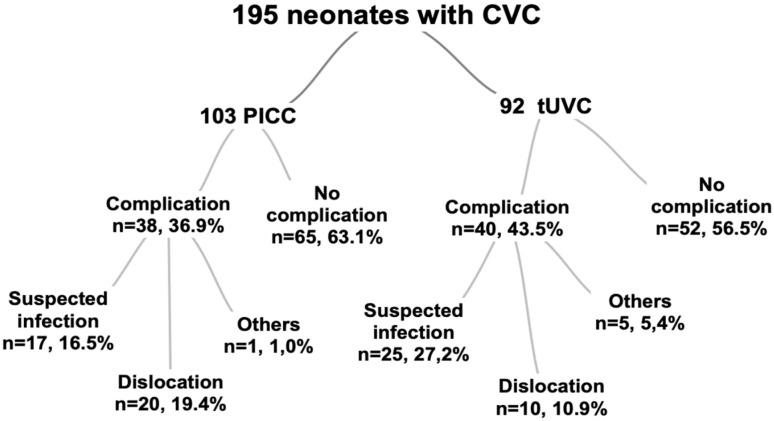
Study population. Abbreviations: CVC, central venous catheter; PICC, peripherally inserted central catheter; tUVC, thin (1-2F) umbilical vein catheter.

The population of this retrospective study included 232 consecutive VLBW infants (birth weights of 1500 g or below) who were born in Tampere University Hospital during the 2011-2016 period and had received a tUVC or a PICC as the first CVC. Approximately 5200 deliveries and 60 to 70 VLBW infants are treated in this tertiary center per year. Infants who were transferred to another hospital (N = 19) or died (N = 18) before the removal of the first CVC were excluded. One of the excluded infants died of sepsis due to an unknown cause, 1 died due to a suspected infection, and the remainder died due to prematurity-related reasons. No deaths were caused by clearly catheter-related factors. To enable a comparison between tUVCs and PICCs, only the first CVC episode was analyzed. The final population included 195 infants (Figure 1). No significant differences were detected between included and excluded infants in terms of birth weight or gestational age (data not shown). The study was approved by the ethical committee of the Pirkanmaa Hospital District.

Information was collected from patient records (Table 1). Chorioamnionitis was considered to be an obstetric clinical diagnosis, as defined previously.^14^ Infants with birth weights below the 10th percentile, after adjusting for gestational age, were considered small for gestational age (SGA).^15,16^ A diagnosis of necrotising enterocolitis (NEC) was made according to the Bell criteria (at least stage 2) or intraoperative biopsy.^17^

**Table 1.** Clinical Characteristics of Infants With and Without Complications in Their First Thin Central Venous Catheter.

	Infants with catheter complications (n = 78)	Infants without catheter complications (n = 117)	*P* value
Birth weight, g, Md (range)	847.5 (520-1385)	1020 (475-1495)	.001
SGA	16 (20.5%)	19 (16.4%)	.46
Gestational age at birth, weeks, Md (range)	27.3 (23.0-32.7)	28.1 (22.7-32.7)	.011
Male sex	40 (51.3%)	65 (55.6%)	.56
Chorioamnionitis	1 (1.3%)	6 (5.1%)	.16
Cesarean section	59 (75.6%)	88 (75.2%)	.95
Multiple birth	25 (32.5%)	37 (31.6%)	.95
Apgar 5 min < 4	11 (14.1%)	11 (9.4%)	.31
Catheter type—tUVC	40 (51.3%)	52 (44.4%)	.35
Infant’s age at catheter insertion, Md (range)	1 (0-32)	1 (0-16)	.14
Catheter dwell time, days, Md (range)	10 (1-59)	13 (1-51)	.003
Number of peripheral iv or arterial lines during catheter dwell time, Md (range)	7 (2-23)	7 (2-31)	.38
Ventilator therapy	58 (74.4%)	67 (57.3%)	.015
Duration of ventilator therapy, days, Md (range)	5.5 (0-61)	2 (0-34)	.004
Duration of parenteral nutrition, days, Md (range)	22 (9-133)	18 (8-94)	.002
Duration of the first antibiotic treatment, Md (range)	5 (0-17)	4 (0-15)	.138
Necrotising enterocolitis	15 (19.2%)	11 (9.4%)	.048

Figures are N (%) unless expressed otherwise. Statistical differences were tested via chi-square test, Fisher exact test and Mann-Whitney *U* test when appropriate.

Abbreviations: Md, median; SGA, small for gestational age, (birth weight for gestational age below the 10th percentile^14^); tUVC, thin (1-2F) umbilical vein catheter.

A PICC or tUVC was inserted if the expected duration of parenteral nutrition was at least 1 week. It was usually removed when enteral feeding at 130 ml/kg/day was achieved. After removal, the catheter tip was sent for a bacterial culture. During the study period, tUVC was preferred as the first CVC in VLBW infants over PICC. The UVC was inserted as described by Haumont^7^ and covered with gauze and a transparent medical dressing. The PICC was usually inserted into a cubital vein using an introducer and then covered with a transparent medical dressing. The catheter tip position was verified by X-rays with contrast media, with the optimal position being extracardial in the central vein, which was confirmed by a pediatric radiologist. Tip positions were re-checked when X-ray or echocardiography examinations were performed due to clinical indications.

Aseptic principles were followed in the handling of the catheters. Checklists for the insertion and maintenance of the catheters were implemented during 2015, and since then, the practice has been to remove or change thin CVCs after 14 days of dwell time if possible. A multidisciplinary group supervised the hygienic procedures in the ward.

Our primary outcome measure was catheter complications, which were defined as non-elective catheter removal for a catheter-related reason more than 24 hours after insertion. Complications were classified as follows: suspected infections; dislocations; and other complications, such as disconnected, blocked, or broken catheters.

The time label for catheter infection was set to the timepoint when the sample for the blood culture was obtained and antibiotic treatment was initiated. For the subgroup analysis of catheter infections, we used 2 time periods: (1) within 1 day before and 1 day after catheter removal and (2) within 3 days before and 3 days after catheter removal. The diagnostic criteria for true catheter infection verification included a positive blood culture without any other focus, as well as the symptoms and signs of infection, such as temperature instability, hypotension, apnea, an increased need for oxygen and/or ventilatory support, heart rate abnormalities, feeding intolerance, lethargy, irritability, skin lesions, and at least one of the following laboratory parameters: white blood cell count < 5 or > 30 x 10^9^ cells/L, immature to total neutrophil ratio > 0.2, absolute neutrophil count < 1000/μL, platelet count < 100 x 10^9^/L, CRP > 15 mg/L, glucose intolerance or hypoglycemia, or acidosis (modified from other studies^18,19^).

### Statistical Analysis

Group comparisons involving categorical variables were performed using cross-tabulation and the chi-square or Fisher exact test, and those involving continuous variables were performed using a Mann-Whitney *U* test. Differences between catheter types in terms of the frequencies of complications and true catheter infections were also analyzed per 1000 catheter days using the OpenEpi program’s Mid-p procedure.

Risk factors were analyzed using univariable and multivariable logistic regression analysis separately for (1) any catheter complication and (2) true catheter infection diagnosed within 3 days before or after catheter removal. The independent variables were gestational age at birth (weeks), SGA (no/yes), gender (female/male), mode of delivery (vaginal/cesarean section), need for mechanical ventilation (no/yes), and central venous catheter type (tUVC/PICC). Gestational age and ventilator therapy were included in the models based on the results of univariable analyses. Also, SGA was included to take into account birth weight, and gender and mode of delivery were included because these are factors known to affect neonatal outcomes.

*P* values < .05 (2-sided) were considered to be statistically significant. Statistical analyses were performed using SPSS Versions 24 and 25.

## Results

### Catheter Complications

Altogether, 92 (47%) infants underwent tUVC insertion, and 103 (53%) infants underwent PICC insertion. In total, 78 (40%) infants underwent the non-elective removal of their first catheter. Of these, 42 (54%) were removed due to suspected infection, 30 (38%) were removed due to dislocation, and 6 (7.7%) were removed for other reasons (3 disconnected, 2 broken, and 1 obstructed catheters; Figure 1). The median catheter dwell time at non-elective removal was 10 days.

The clinical characteristics of the infants are presented in Table 1. Infants who experienced complications regarding their first catheter had lower birth weights, were born more prematurely, and needed ventilator therapy more commonly and for longer periods as compared with those who did not experience complications.

### Comparison Between Catheter Types

The comparisons between infants with tUVCs and those with PICCs are presented in Table 2. Umbilical catheters were inserted at an earlier age than PICCs (median (range): 0 (0–5) vs 2 (0-32) days, *P* < .001). The most common reasons for non-elective catheter removal were suspected infection in the tUVC group and dislocation in the PICC group. No significant difference was found in the total catheter complication rate between infants with tUVCs and those with a PICC. True catheter infection within ±3 days of catheter removal was more common in the tUVC group than in the PICC group, but the groups did not differ in terms of the ±1 day criteria. The occurrence of NEC was similar between the groups (tUVC 12 (13.0%) vs PICC 14 (13.6%), *P* = .910).

**Table 2.** Comparison Between Infants With Thin Umbilical Catheters and Those With Peripherally Inserted Central Venous Catheters, *N* = 195.

	tUVC, n = 92	PICC, n = 103	*P* value
Birth weight, g, Md (range)	867.5 (520-1490)	1050 (475-1495)	<.001
SGA	11 (12.1%)	24 (23.3%)	.043
Gestational age at birth, weeks, Md (range)	26.7 (22.7-31.9)	28.3 (23.6-32.7)	<.001
Male sex	49 (53.3%)	56 (54.4%)	.88
Chorioamnionitis	5 (5.4%)	2 (1.9%)	.19
Cesarean section	71 (77.2%)	76 (73.8%)	.58
Multiple birth	23 (25.0%)	39 (37.9%)	.054
Apgar 5 min < 4	8 (8.7%)	14 (13.6%)	.28
Catheter dwell time, days, Md (range)	12 (1-32)	13 (1-59)	.061
Any catheter complication N/1000 catheter days	40 (43.5%) 37.5/1000	38 (36.8%) 26.3/1000	.35 .12
Reason of non-elective removal			
Suspected infection N/1000 catheter days	25 (27.2%) 23.4/1000	17 (16.5%) 11.8/1000	.070 .028
Dislocation N/1000 catheter days	10 (10.9%) 9.4/1000	20 (19.4%) 13.9/1000	.099 .32
Others N/1000 catheter days	5 (5.4%) 4.7/1000	1 (1.0%) 0.69/1000	.10 .060
True catheter infection^a^ (±1 days) N/1000 catheter days	12 (13.0%) 11.2/1000	9 (8.7%) 6.2/1000	.33 .19
True catheter infection^a^ (±3 days) N/1000 catheter days	19 (20.7%) 17.8/1000	12 (11.7%) 8.3/1000	.086 .038
Positive catheter tip culture	31 (54.4%) n = 58	31 (38.3%) n = 81	.076
Duration of parenteral nutrition, days, Md (range)	20 (8-109)	19 (8-133)	.23

Figures are N (%) unless expressed otherwise. Statistical differences were tested by chi-square test, Fisher exact test, Mann-Whitney *U* test and OpenEpi program’s Mid-p procedure when appropriate.

Abbreviations: tUVC, thin (1-2F) umbilical vein catheter; Md, median; PICC, peripherally inserted central catheter; SGA, small for gestational age.

a Positive blood culture + symptoms/signs of infection.

Ten infants died during their hospital stays after the removal of the first catheter. There were more deaths in the tUVC group than the PICC group (8 (8.7%) vs 2 (1.9%), *P* = .033). In 2 cases in which tUVCs were inserted, the cause of death was catheter sepsis. Eight deaths did not have catheter-related causes and occurred 4 to 44 days after the removal of the first catheter.

### Risk Factors for Catheter Complications and Infections

The results of the risk factor analyses for catheter complications generally are presented in Table 3. In the univariable analysis, low gestational age and ventilator therapy were associated with catheter complications. A multivariable analysis showed no significant association between ventilator therapy and catheter complications, and the OR (95% CI) for the association between gestational age and catheter complications was 0.822 (0.675-1.000, *P* = .05).

**Table 3.** Logistic Regression Analysis of Risk Factors for Having Any Catheter Complications (N = 78) or True Catheter Infection Within ±3 Days of Catheter Removal (N = 31).

Independent variables	Any complication						True catheter infection^a^					
	Univariable analysis			Multivariable analysis			Univariable analysis			Multivariable analysis		
	OR	95% CI	*P*	OR	95% CI	*P*	OR	95% CI	*P*	OR	95% CI	*P*
Gestational age (weeks)	0.847	0.735-0.976	.022	0.822	0.675-1.000	.05	0.949	0.920-0.979	.001	0.941	0.900-0.984	.008
SGA	1.317	0.630-2.754	.464	2.410	0.970-5.987	.058	1.175	0.424-3.253	.757	3.265	0.858-12.421	.083
Male gender	0.842	0.474-1.496	.558	0.859	0.462-1.599	.633	0.744	0.336-1.650	.467	0.737	0.302-1.800	.503
Cesarean section	1.023	0.526-1.992	.946	0.903	0.429-1.900	.788	0.947	0.381-2.355	.907	0.953	0.323-2.810	.930
Ventilator therapy	2.164	1.157-4.049	.016	1.876	0.858-4.099	.115	3.190	1.215-8.378	.019	1.394	0.416-4.666	.590
Number of other peripheral intravenous or arterial lines during catheter dwell time,	0.996	0.937-1.060	.910	0.959	0.893-1.031	.255	1.056	0.973-1.147	.190	0.997	0.899-1.105	.951
CVC catheter type (tUVC)	1.316	0.741-2.337	.349	1.042	0.548-1.982	.900	2.068	0.917-4.662	.080	1.543	0.612-3.891	.358

Abbreviations: OR, odds ratio; CI, confidence interval; SGA, small for gestational age; CVC, Central venous catheter; tUVC, thin (1-2F) umbilical vein catheter.

a Positive blood culture within 3 days before or after catheter removal + symptoms and signs of infection.

In total, 21 infants (10.8%, 8.4/1000 catheter days) had true catheter infections (positive blood culture combined with symptoms) within ±1 day of catheter removal, and 31 infants (15.9%, 12.3/1000 catheter days) had such within ±3 days of catheter removal. Among the latter, 26 catheters were removed due to suspected infections, 1 was removed due to catheter obstruction, and 4 were removed electively. In these 5 cases, the signs and symptoms of infection developed within 3 days after catheter removal.

In the univariable analysis, lower gestational age and ventilator therapy were associated with true catheter infection within 3 days before or after catheter removal. However, in the multivariable logistic regression analysis, only lower gestational age remained a significant risk factor for catheter infection. No association was found between catheter infection and CVC type (Table 3).

The most common pathogen in both the blood and catheter tip cultures was coagulase-negative staphylococcus (CoNS). Tip culture findings were available for 139 catheters (71.3%). Of these, 62 (44.6%) revealed pathogen growth, including 30 (35%) of the electively removed catheters and 7 that were removed due to dislocation. The tip culture was positive in 19 of 31 catheters (61.3%) in the true catheter infection group, and for 13 catheters (41.9%), the culture revealed the same bacteria in the blood and on the catheter tip.

## Discussion

In this study of VLBW infants, non-elective removals of the first thin umbilical CVCs and peripherally inserted CVCs were common. Nearly half of removals were due to suspected infection. We did not find significant differences in total complication rates between tUVCs and PICCs, but non-elective removals due to suspected infection were more common in the tUVC group. Two infants with tUVCs died due to catheter infection. Also, the rate of infections/1000 catheter days was higher in the tUVC group than in the PICC group. However, in the multivariable logistic regression analysis, the only statistically significant risk factor for true catheter infection was low gestational age.

Previously, non-elective PICC removal rates in neonates^8,20-22^ and infants^23^ have been found to vary from 9.97% to 37.2%. In our study, a high percentage (40%) of the first thin CVCs, which were either PICCs or tUVCs, were removed non-electively. Differences in patient populations and the definitions of complications mostly account for the differences between studies. We did not find any significant differences between the overall complication rates for tUVCs and PICCs. Previous studies have investigated mainly traditional, uncovered UVC catheters and had various study designs, so they are not comparable with our study.^2,8^ To our knowledge, this study is one of the few focusing on premature VLBW infants and the use of tUVCs.

As in prior studies, infants with any catheter complications had lower birth weights and gestational ages than those without complications,^13,20,21,23^ as well as having longer parenteral nutrition periods.^13^ They were also more commonly exposed to other invasive treatments, including ventilator therapy, and such treatments lasted longer. We focused on complications regarding the first thin CVCs, and no strict conclusions can be drawn regarding their role in the development of later problems, such as NEC.

In our population, infants with tUVCs were lighter and more premature at birth than infants with PICCs, which may have contributed to the differences in infection and death rates. The higher percentage of deaths in the tUVC group was likely not associated with the use of tUVCs. A multivariable logistic regression analysis did not reveal any association between catheter type and the risk of either any catheter complications or catheter infection. Thin UVCs have some benefits as compared with PICCs because they can be inserted without pain and without penetrating the skin of a fragile infant. When tUVCs are used as the first CVCs, the peripheral veins remain intact for later use, which is beneficial, especially when the need for prolonged parenteral nutrition is likely.

Our definitions of “catheter-related sepsis” were based on positive blood cultures and symptoms and laboratory findings compatible with infection within 1 or 3 days before or after catheter removal. The removal of CVCs has been suggested to be associated with bacteremia within 72 hours after catheter removal, even in 23% of the sepsis cases.^24^ We included this wider timeframe to ensure that no catheter-related infections would be missed. This shorter timeframe is in accordance with Center for Disease Control and Prevention criteria.^25^

Previous studies have reported sepsis rates of 1.66-18.1/1000 catheter days.^10-13,26,27^ Our infection rates were within this range. The various hygienic practices of the units, ward patient loads, and patient case mixes help explain the differences between studies. According to our logistic regression analysis, the most important risk factor for catheter infections was low gestational age, as reported previously.^21,26^ In very low gestational age infants, an increased need for invasive treatments, fragile skin barrier, and immature immune system contribute to an increased risk of infections.^28^

In line with previous studies, CoNS was the most common pathogen found in the blood^6,11,26,27,29^ and catheter tip cultures.^21^ Asymptomatic bacterial contamination of the catheters was common.^30^ The use of catheter tip cultures as diagnostic criteria for catheter-related sepsis is challenging. The infant may have received several doses of antibiotics, sometimes via the CVC, before the removal of the catheter, which may bias the culture results.

The limitations of this study were its retrospective design and relatively small sample size. However, our results add to the knowledge about CVC complications in VLBW infants. The catheter-related sepsis classification system we used was clinically applicable. Because all infants were treated in one unit, we had access to all clinical details in the patient records. Treatment practices did not differ markedly between patients.

## Conclusion

Gestational age was associated with both central venous catheter complications and infections in VLBW infants. No clear association was found between central venous catheter type and catheter complications. Our data suggest that neither tUVCs nor PICCs are superior regarding complication risk.

## Author Contributions

SS had primary responsibility for data collection, data analysis and writing the manuscript and contributed to the development of the protocol and literature search. OT participated in the development of the protocol and analytical framework for the study and contributed to the writing of the manuscript and literature search. AMK contributed to the data analysis and to the writing of the manuscript. PK had primary responsibility for the development of the protocol and analytical framework for the study and participated in literature search, data collection, data analysis and writing of the manuscript.
